# Tipping the Balance: *C. albicans* Adaptation in Polymicrobial Environments

**DOI:** 10.3390/jof4030112

**Published:** 2018-09-18

**Authors:** Amit Ranjan, Anna Dongari-Bagtzoglou

**Affiliations:** Department of Oral Health and Diagnostic Sciences, School of Dental Medicine, University of Connecticut, Farmington, CT 06030, USA; aranjan@uchc.edu

**Keywords:** polymicrobial interactions, *Candida albicans*, candidiasis, fungal-bacterial interactions

## Abstract

*Candida albicans* is a pleiomorphic fungus which co-exists with commensal bacteria in mucosal and skin sites of mammalian hosts. It is also a major co-isolated organism from polymicrobial systemic infections, with high potential for morbidity or mortality in immunocompromised patients. Traditionally, resident mucosal bacteria have been thought to antagonize *C. albicans* in its ability to colonize or cause infection. However, recent investigations have revealed synergistic relationships with certain bacterial species that colonize the same mucosal sites with *C. albicans*. Such relationships broaden the research landscape in pathogenesis but also contribute to clinical challenges in the prevention or treatment of mucosal candidiasis. This review sheds light on interactions of *C. albicans* and mucosal bacteria, with special emphasis on the effects of the resident bacterial microbiota on *C. albicans* physiology as they relate to its adaptation in mucosal sites as a commensal colonizer or as a pathogenic organism.

## 1. Introduction

There is ample evidence for cross-kingdom signaling, cross-regulation, antagonism, and synergy between bacteria and *C. albicans*. As we acquire more information on the composition of microbiota residing in mammalian mucosal tissues using advanced sequencing methodologies, it is imperative to begin to examine how organisms colonizing the same ecological niches modulate the capacity of each other to colonize or infect the host, and how the host regulates homeostasis under these conditions. *C. albicans* is a fungal organism which can switch from commensal to pathobiont, and is associated with the majority of mucosal infections [1,2]. This review focuses on the effects of the resident bacterial microbiota on *C. albicans* physiology as they relate to its adaptation in mucosal sites as a commensal colonizer or pathogenic organism.

## 2. Mucosal Microbiota Act as an Innate Host Factor That Controls the Switch from Fungal Commensalism to Infection

### 2.1. Effect of Indigenous Bacteria in Mammalian Host Colonization Models

*C. albicans* colonizes several mammalian polymicrobial host niches, including oropharyngeal, esophageal, vaginal, and gastrointestinal mucosae, and the skin. In each of these sites it has to compete for space and nutritional resources with other commensal fungi and bacteria and respond to environmental cues that promote a steady state of commensalism or lead to infection. It is widely accepted that in a healthy host, the resident bacteria play an important role in limiting the capacity of *Candida* for colonization. This may be accomplished by direct fungal–bacterial cell interactions involving secreted bacterial products. For example, certain *Enterococcus faecalis* strains secrete a bacteriocin that promotes a non-filamentous, commensalism-associated phenotype of *C. albicans* in nematodes and orally inoculated mice [3,4]. In a similar function, a secreted protein of *Salmonella enterica* attenuates the virulence of *C. albicans* in *C. elegans* and promotes a symbiotic state in the nematode gut [5,6]. Alternatively, resident microbiota may act indirectly to inhibit colonization by enhancing the mucosal immune response to *Candida*. An example of this is *Corynebacterium mastitidis*, a member of the resident bacterial microbiota in the murine ocular mucosa, which prevents *C. albicans* infection by driving IL-17 production from mucosal T cells [7]. Along the same line, *Bacteroides thetaiotaomicron*, a commensal of the murine intestinal mucosa, prevents *C. albicans* colonization by stimulating the epithelial antimicrobial proteins cathelicidins [8].

Several studies have examined the role of indigenous bacteria in alimentary-tract colonization of healthy mice using combinations of broad-spectrum antibiotics and monitoring the growth of both bacteria and *Candida* in the post-antibiotic fungal colonization period. Enhanced oropharyngeal and intestinal colonization of *C. albicans* was noted with the most broad-spectrum antibiotics in mice [8,9,10,11]. Interestingly, antibiotics, and by extension, indigenous bacteria which are either displaced or overgrown with antibiotic treatment can also affect the outcome of competition between *C. albicans* and *C. glabrata* in mouse cecum [12]. It is well-established that commensal anaerobic bacteria are critical in limiting *Candida* intestinal colonization in mice, and colonization levels are generally proportional to the level of antibiotic depletion of anaerobic bacteria [13]. On the other hand, antibiotics that allow *Escherichia coli* overgrowth inhibit murine cecum colonization [12]. Along these lines, an increase in *Enterococcus* species in the stomach [10], caecum [11], and terminal ileum [9], and *Streptococcus* species in the colon [9] in the post-antibiotic period were associated with increased *C. albicans* colonization. On the other hand, lactobacilli antagonize *C. albicans* colonization in lower GI-tract mucosa [9,11], although their role in the oropharyngeal mucosa has not been established in vivo. Using innovative predictive statistical models, Shankar and colleagues [9] showed greater dependence of *Candida* colonization in the murine ileum and colon on certain bacterial genera than the host cytokine environment in the two sites. These studies revealed that certain bacterial genera (e.g., *Veillonella*) may influence *C. albicans* colonization by modulating the GI immune response, whereas others (e.g., *Streptococci*) may have a direct impact on *Candida* physiology that does not involve the intestinal immune response [9].

### 2.2. Effect of Indigenous Bacteria in Mucosal Candidiasis

A central tenet in the pathogenesis of mucosal candidiasis is that immunosuppression, or other mucosal barrier-compromising conditions (e.g., local trauma or inflammation) are a prerequisite for infection [14,15]. Under inflammatory or immunocompromised host conditions, it is possible that ecological resources, such as space and nutrients, are diverted to favor bacterial species that form mutualistic relationships with *C. albicans*. This, in turn, could lead to a well-coordinated fungal-bacterial dysbiosis which amplifies mucosal damage. Such pathogenic synergy between *C. albicans* and certain commensal bacterial species was recently revealed in mouse models of infection. For example, in cortisone-immunosuppressed mice, we showed that when *C. albicans* is orally co-inoculated with mitis-group streptococcal species, there is an increase in oral mucosal biofilms that contributes to *Candida* virulence [16]. Others have shown pathogenic synergy between *C. albicans* and *Staphylococcus aureus* in a similar oral infection model [17]. A peritonitis model from the Noverr laboratory has demonstrated that rates of disease progression and microbial load in mice infected with both *S. aureus* and *C. albicans* were significantly higher compared to those with monomicrobial infections [18]. In both the oropharyngeal and peritonitis infection models, pathogenic synergy between Gram (+) cocci and *C. albicans* was shown to be host-response mediated via induction of a significantly higher proinflammatory response [16,18]. In the peritonitis model, pathogenic synergy was primarily eicosanoid-mediated [18], whereas in the oropharyngeal model, an exaggerated TLR-2-dependent chemokine and neutrophil response was involved [16]. Similarly, in a zebra fish swim bladder infection model which emulates the human lung mucosa, *Pseudomonas aeruginosa* promoted *C. albicans* virulence, associated with higher fungal invasion, proinflammatory cytokine production, and immunopathology [19]. These studies were performed with exogenously provided bacteria, and a role for indigenous communities or dysbiosis in fungal pathogenesis has not been examined thus far at any mucosal site. A possible exception to this is the murine gastric mucosa where indigenous enterococci were indirectly suggested to be implicated in the pathogenesis of *Candida*-induced gastritis [10]. 

Disease-promoting interactions between *Candida* and resident mucosal bacteria may be mucosal site-specific. For example, mice pretreated with antibiotics and then inoculated with *C. albicans* experience significant inflammatory and ulcerative gastric lesions, but do not develop signs of inflammation in the cecum, despite having similar fungal burdens at this site [10,11]. Similarly, in the lower GI tract, Gram (-) anaerobes and enteric bacilli limit the growth and dissemination of *Candida* [20], and after treatment with broad-spectrum antibiotics, *C. albicans* can invade the gut mucosa [21,22]. In contrast, reports of oropharyngeal candidiasis or oral mucosal invasion after similar antibiotic treatment are rare [23]. Thus, the effect that indigenous bacteria have on mucosal candidiasis is likely to differ at different mucosal sites, due in part to the anatomically distinct innate immune epithelial response to infection, but also due to site-specific differences in the composition of the local microbial communities [24].

A high percentage (>60%) of *C. albicans* nosocomial and immunocompromised patient infections are polymicrobial, and numerous clinical studies have reported co-isolation of bacteria from human disease samples (reviewed by O’Donnel et al. [25]). For example, *P. aeruginosa*, *S. aureus*, and streptococci of the mitis group are frequent co-isolates with *C. albicans* in neonatal bloodstream infections and pulmonary infections in antibiotics-treated immunocompromised patients [26,27]. There is also mounting evidence that *C. albicans* and enterococci co-exist in human disease samples (reviewed by Garsin and Lorenz, [28]). Perhaps the strongest evidence comes from a large-scale retrospective analysis showing that *E. faecalis* was twice as likely to be isolated in *Candida*-positive sputum or skin samples and even more likely in sepsis [29]. In the oral cavity, *E. faecalis* and *C. albicans* were co-isolated in >10% of root canal infections [30] and in >40% of tongue mucosal lesions in humans [31]. Whether this co-isolation reflects similar mucosal adaptation strategies or local dysbiotic shifts which contribute to pathogenic synergy remains unclear. Most human clinical studies which have identified both *C. albicans* and bacteria in disease samples are cross-sectional and observational, and cannot causally link these organisms to disease progression or prevention. No study to date has directly or systematically assessed the role of indigenous bacteria in the pathogenesis of mucosal candidiasis in humans.

## 3. Bacterial Signals Trigger Distinct Phenotypic Responses in *C. albicans* Associated with Commensalism or Virulence

### 3.1. Effect of Bacterial Interactions on Filamentation

Because *C. albicans* has to adapt in polymicrobial mucosal environments, it is not surprising that it responds to bacterial signals with distinct phenotypic changes. The most readily identifiable phenotypic response to these signals is their effect on hyphal morphogenesis. In vivo indirect evidence supporting the fact that *C. albicans* responds to indigenous microbiota by activating filamentation pathways comes from works with germ-free mice. In these mice, *C. albicans* exists in the lower GI tract preferentially in the yeast form, whereas a mix of hyphae and yeast cells colonize the same sites in conventional mice [32]. In contrast, *C. albicans* forms highly invasive hyphae on the tongues of germ-free piglets independently of the Efg1 filamentation pathway, since deletion of *efg1* did not abrogate filamentation at this site [33]. To complicate matters further, recent work from our laboratory showed activation of the Efg1 filamentation pathway in *C. albicans* by mitis-group streptococci, not indigenous in the mouse oral cavity, leading to more invasive oral infection in a murine oropharyngeal candidiasis model [34,35]. These observations raise the possibility that resident bacteria modulate the ability of fungal cells to form tissue-invasive hyphae in a mucosal site- and local microbiota-specific manner. 

Interactions between *C. albicans* and bacteria that affect filamentation may be contact-dependent or mediated by secreted bacterial signals. Most published studies have focused on bacterial secreted products that inhibit the hyphal switch because of their translational potential in preventive or therapeutic applications. Secreted signals include N-acyl homoserine lactones, the primary quorum sensing molecules in Gram (-) bacteria produced when the cell density is high. One of these molecules produced by *P. aeruginosa* inhibit the yeast-to-hyphae switch [36], but hypoxic conditions dampen its production and limit its effect on filamentation [37], highlighting the fact that environmental conditions, such as site-specific oxygen tension, may modify the outcome of these interactions. Autoinducer-2, another secreted bacterial quorum-sensing molecule, has been reported to be involved in both enhancing [38] and suppressing filamentation [39], depending on the bacterial species—however, the exact mechanisms are not completely understood. Pyocyanin, produced by *P. aeruginosa* in the early stationary phase, inhibits the switch by reducing intracellular cAMP, high levels of which signal the transition from yeast to hyphae [40]. Streptococcal competence-stimulating peptides may also disrupt hyphal morphogenesis, although this effect appears to be streptococcal species-dependent [41,42]. Finally, a secreted bacteriocin by *E. faecalis*, which is partially dependent on the Fsr quorum-sensing pathway, inhibits the switch to hyphae [3]. 

Bacterial cell wall components, such as peptidoglycan fragments and LPS, have been shown to exert opposing effects, with the former inducing [43] and the latter decreasing [44] filamentation. Hydrogen peroxide, a major metabolic byproduct released by actively growing streptococci, although genotoxic in high concentrations, can stimulate filamentous growth in *C. albicans* at lower concentrations [45,46]. Early work by Noverr and Huffnagle [47] demonstrated that several live *Lactobacillus* species inhibit *C. albicans* hyphal transformation via soluble metabolic products and possibly short-chain fatty acids, such as butyric acid. By significantly reducing the environmental pH, lactic acids produced by lactic-acid bacteria (e.g., streptococci, enterococci, lactobacilli) have the ability to negatively influence filamentation, although under glucose-limiting conditions, *C. albicans* can neutralize their effect by excreting ammonia [48]. Contact-dependent signals with a detrimental effect on hyphae have also been identified in certain Gram (-) bacteria (e.g., *Acinetobacter baumannii*) [49].

### 3.2. Effect of Bacterial Interactions on C. albicans Metabolism, Growth, and Biofilm Formation

Metabolic interactions within polymicrobial communities in mucosal sites play a major role in the adaptation of each community member to these sites. In support of a possible role of indigenous intestinal bacteria in fungal metabolism, a recent study by Noble et al. has shown that the passage of *C. albicans* through the murine gastrointestinal (GI) tract induces a fungal-cell phenotype termed “GUT”, which differs both morphologically (elongated yeast cells) and metabolically from cells growing in other conditions [50]. In particular, these fungal cells express transcriptome-enriched genes involved in the catabolism of bacterial products, such as fatty acids and *N*-acetylglucosamine. Work from the Kumamoto lab also showed that hypercolonization in the cecum by certain mutant *C. albicans* strains was associated with an increased ability to utilize non-fermentable carbon sources such as short chain fatty acids, produced by *Bacteroides* in the lower GI tract [51]. Therefore, it is tempting to speculate that intestinal bacteria contribute to the metabolic adaptation and growth of *C. albicans* in the intestinal mucosa, although direct comparisons in gnotobiotic mice were not performed in these studies.

More direct evidence for the role of bacteria in influencing *C. albicans* metabolism comes from in vitro studies. *L. ramnosus* growing with *C. albicans* on epithelial monolayers in vitro revealed metabolic reprogramming in *C. albicans*, primarily by induction of fatty acid metabolism, glyoxylate pathway-, and gluconeogenesis pathway-related genes, while the glucose metabolic pathway was downregulated. This may promote growth in mucosal sites rich in commensal lactobacilli, such as the intestinal and oral mucosa, under conditions of nutritional compromise or lower glucose availability [52]. A recent transcriptomic analysis of the *C. albicans* response to *S. gordonii*, another oral commensal, revealed that biosynthetic genes involved in arginine production were enriched in mixed biofilms, whereas transcription of the arginase gene (*car1*) was downregulated [53]. This may represent a fungal cell response to high amounts of H_2_O_2_ produced by this streptococcal species [54,55].

In mixed biofilms with *P. aeruginosa* in vitro, *C. albicans* exhibits lower metabolic activity with a decrease in iron-dependent processes due to increased iron sequestration by bacteria. In these studies, increased secretion of a bacterial-specific siderophore has been suggested to decrease availability of iron to fungal cells and reduce their metabolic activity [56]. Glycolysis is also decreased in *C. albicans* biofilms treated with high amounts of *P. aeruginosa* LPS [44].

Since hyphal growth is a main feature in mature biofilms, in theory, any bacterial signal that promotes or inhibits filamentation can alter the ability of *C. albicans* to grow in the biofilm form. Thus, it is not surprising that acyl-homoserine lactones from *P. aeruginosa* have anti-biofilm activity and cause dispersal of yeast cells from biofilms [57]. In contrast to *P. aeruginosa*, several oral streptococcal species have been reported to enhance *C. albicans* biofilms by promoting hyphal growth. In particular, mitis-group streptococci, such as *S. oralis* and *S. gordonii*, form co-aggregation interactions with hypha-associated cell wall adhesins, such as Als1 and Als3, which increase biofilm biomass [16,58,59]. On the other hand, oral viridans streptococci, principally represented by *S. mutans*, promote fungal biofilm formation on tooth surfaces that are normally not colonized by *C. albicans* by depositing bacterial extracellular polysaccharides (α-glucans) on the surface of fungal cells, which promote co-aggregation interactions [60].

## 4. Bacteria Influence *C. albicans* Gene Expression and Site-Specific Adaptation in Mammalian Host Niches

*C. albicans* responds rapidly to environmental conditions in different mucosal sites. Mucosal-site-specific differences in fungal gene expression are expected due to the requirement for adaptation in environments with different mucosal physiology, but also with different indigenous bacterial microbiota. For example, upregulation of oxidative stress genes (e.g., *trx1*, *cat1*) may be more important in upper alimentary-tract mucosal adaptation since the dominant streptococcal and lactobacilli taxa in this site can generate cytotoxic levels of H_2_O_2_ [55]. Gene expression changes during adaptation can be monitored locally at the transcriptional level by in vivo gene expression profiling to identify functional adjustments of the fungus within polymicrobial communities. Two studies have examined the *C. albicans* transcriptome in situ, in mucosal niches that harbor diverse indigenous bacterial communities (i.e., intestinal and vaginal mucosa) [51,61]. However, neither study compared fungal transcriptomic profiles at the same sites in conventional versus gnotobiotic mice, therefore a direct or indirect influence of indigenous commensals on *C. albicans* gene expression during adaptation in these sites was not examined.

Although the role of bacteria was not directly addressed, a microarray analysis of *C. albicans* gene expression during post-antibiotic murine intestinal colonization has provided some insights by comparing the transcriptional responses during adaptation in the ileum and cecum of the same mice [51]. The two sites differ by the considerably higher presence of bacteria and their short-chain fatty acids in cecum compared to ileum. Only approximately half of the differentially regulated genes in the two sites overlapped, and these included genes involved in host interactions, pathogenesis, and metabolism. Interspecies-interaction genes were not identified in the overlapping gene ontology categories, reflecting differences in bacterial environments. In the murine vaginitis model, RNAseq analysis of the most highly expressed genes of *C. albicans* revealed certain genes associated with interspecies interactions (e.g., *sod5*, involved in oxidative responses)—however, the role of vaginal commensal bacteria, such as lactobacilli, which can induce oxidative stress in *C. albicans* by releasing H_2_O_2_ was not examined [61].

Most studies on the effects of bacteria on *C. albicans* gene expression have used in vitro models. For example, the influence of mixed biofilm growth with enteric bacteria on *C. albicans* global gene expression was studied in biofilms growing on polystyrene surfaces using microarray analysis. Strongly upregulated by coculture with *Klebsiella pneumoniae*, *E. coli* and *E. faecalis* were the “master” regulator of white–opaque-switching WOR1, which, however, did not allow a complete switch from the white to opaque phenotype [62]. Two other studies independently confirmed that this regulator was also upregulated by the intestinal mucosal environment in vivo [50,51]. This raises the possibility that upregulation of WOR1 and at least partial induction of this phenotypic switch is an adaptive response of *C. albicans* to the exposure to enteric commensals in the intestinal milieu.

Several studies have shown upregulation of virulence-associated genes by certain bacterial species. Gene-expression analysis of *C. albicans* forming polymicrobial biofilms with oral bacteria on epithelial constructs in vitro showed that known virulence genes encoding certain secreted aspartyl-proteinases (*sap4*, *sap6*) which facilitate fungal invasion [63] were up-regulated [64]. In fact, using an oropharyngeal candidiasis model, we showed that co-inoculation of mice with *S. oralis* caused increased expression of *sap4*, *sap5*, and *sap6* genes and also increased systemic dissemination, implicating commensal bacteria in enhanced virulence [34]. More recently, RNAseq analyses of *C. albicans* forming biofilms with *S. gordonii* in vitro showed filamentation- and adhesion-related genes involved in pathogenesis and virulence being upregulated (e.g., *als1, tec1*) [53]. Other genes included *hyr1*, which may protect *C. albicans* from neutrophil damage during adaptation in mucosal sites [65].

## 5. *C. albicans* Has Reciprocal Effects on the Mucosal Bacterial Microbiota

The influence of *C. albicans* on the resident bacterial composition at mucosal sites has seldom been addressed in the literature, and most studies investigating this aspect of fungal pathogenesis were conducted in the murine intestinal mucosa. Low-level colonization of the murine cecum of healthy C57B/L6J mice by *C. albicans* led to shifts in the community structure, such that bacterial communities in the colonized mice were distinct from and more diverse than naive mice [10]. It has been suggested that *C. albicans* non-pathogenic colonization of the lower GI tract in mice may promote the growth of *Bacteroidetes* [11]. Recent evidence has shown that introduction of *C. albicans* in the gastric or intestinal mucosa of antibiotics-treated mice displaces lactobacilli and drives a preferential re-colonization by enterococci [10,11]. In a nematode gut infection model, *C. albicans*-produced farnesol inhibited *A. baumannii* growth [49].

Although ethanol produced by *C. albicans* stimulated in vitro biofilm formation in *P. aeruginosa* [66], results from murine infection models dispute the notion that *C. albicans* aggravates *P. aeruginosa* virulence. For example, *C. albicans* short-term pulmonary colonization prior to *P. aeruginosa* inoculation caused a reduction in the *P. aeruginosa* bacterial load by recruiting natural killer cells and increasing IL-22 in lung tissues [67,68]. Similarly, in a murine intestinal infection model, *C. albicans* secreted factors inhibited the expression of siderophores as well as cytotoxic molecules produced by *P. aeruginosa*, thereby reducing its virulence [69]. 

In oral infection models, the introduction of *C. albicans* to the oral cavity of mice facilitates colonization of mucosal surfaces by *S. oralis*, a streptococcal species that preferentially colonizes tooth surfaces [16]. Hence, just as *S. mutans* creates favorable conditions for tooth colonization by *C. albicans* [70], *C. albicans* creates favorable conditions for mucosal colonization for other streptococcal species that normally colonize teeth [71]. This reciprocal relationship extends to streptococcal infection models with *S. mutans*, where *C. albicans* enhances the cariogenicity of streptococci by promoting expression of the biofilm matrix synthesizing enzyme, GtfB [72]. 

In vitro biofilm experiments have shown that *C. albicans* biofilms create hypoxic or anoxic microniches that allow for the growth of obligate anaerobic bacteria under ambient oxygen tension [62,73]. *C. albicans* inoculated in microcosm biofilms growing directly from saliva can induce growth of anaerobic bacteria under aerobic conditions via mitochondrial oxygen consumption, which causes a micro-aerobic/anaerobic niche [73]. A similar growth advantage was conferred to anaerobic enteric bacteria (*Clostridium perfringens* and *Bacillus fragilis*) by *C. albicans* growing on polystyrene plates [62]. A similar effect was also shown in mixed biofilms with facultative streptococcal species that thrive in lower oxygen tensions [74].

## 6. Unanswered Questions on Fungal-Bacterial Interactions in Human Health and Disease

In recent years, there has been a growing appreciation of the fact that *C. albicans* almost never exists in isolation from other microorganisms in mucosal sites. Thus, the pathogenesis of candidiasis has to be studied both in the context of site-specific immunity and site-specific microbiota. Mouse studies of *C. albicans* have largely focused on its virulence, rather than on its ability to co-exist as a commensal or pathogen in the alimentary tract microbiota. As such, little is known about the microbiome-mediated interactions that control the switch from commensalism to infection. Although several studies have shown pathogenic synergy between *C. albicans* and experimentally inoculated bacteria, very few studies have focused on the interplay between *C. albicans* and indigenous mucosal bacteria in health and disease. 

The examination of fungal adaptation within different polymicrobial mucosal environments in the same hosts using transcriptomic methods has not received significant research attention. Although studies over the past two decades have performed detailed transcriptomic analyses in deep organs which are sterile, a gap in knowledge still exists on the role of specific bacteria in fungal adaptation within different polymicrobial mucosal habitats [75,76]. In addition, the use of antibiotics as part of the fungal infection protocol in oral models to improve fungal burdens has obscured the role of resident bacteria in fungal infection [17]. Although CFU counts increase with antibiotics in the oral mucosa [77], no study has shown that this is accompanied by inflammatory changes unless an immunosuppressive regimen is also administered.

The clinical significance of bacterial pathobionts in fungal pathogenesis is the recalcitrance of most *Candida* infections to antifungals, since current therapies do not address the potential influence of these indigenous bacteria in virulence. Targeting the organism that is crucial for pathogenicity in mixed biofilm infections with antimicrobials is a challenge, although broad-spectrum antimicrobials such as ethanol or chlorhexidine, in theory, may be effective. However, if a bacterial partner is a key factor in increasing *C. albicans* biofilms in host niches that do not otherwise provide fungal adhesion sites, control of infection at these sites may be accomplished solely with an effective antibiotic regimen without the need for an antifungal. Translational studies that can pursue such novel preventive or therapeutic strategies in mucosal *C. albicans* infections are needed.

Several studies have examined the global transcriptome of *C. albicans* colonizing the lower murine GI tract after antibiotics treatment, and some transcriptome studies were performed in kidneys/livers in the intravenous fungal inoculation model [75,76,78,79,80]. However, there are no studies addressing the functional gene adaptation of *C. albicans* in the alimentary tract of mice harboring unperturbed bacterial communities, and no studies are available addressing the oropharyngeal colonization site. One reason for this, and one of the biggest challenges for global fungal transcription analysis in mucosal and other tissues, is the inadequate amounts of fungal RNA recovered amidst a high abundance of mammalian transcripts in these samples. This is especially problematic with eukaryotic pathogens since they share the polyadenylation of mRNA with their hosts, meaning poly-A enrichment protocols cannot enrich fungal transcripts. High-quality gene-expression analysis can be accomplished with 2 million reads per sample for fungi [79,81]. However, this level of coverage is not accomplished even in infected (i.e., not merely colonized) mucosal tissues, as Bruno and colleagues have shown in a murine vaginitis model [61]. More studies with novel fungal gene enrichment strategies are needed to compare the fungal transcriptomic responses in the same mucosal sites in gnotobiotic and conventional mice.

**Figure 1:**
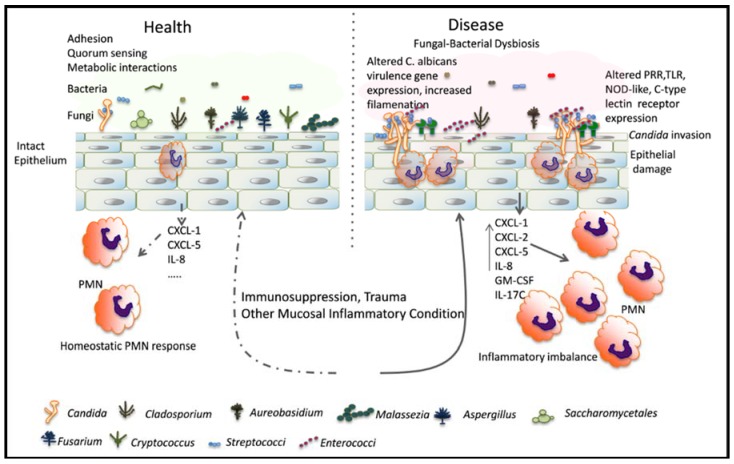
Relationship between *C. albicans* and resident mucosal bacteria in heath and disease: Immunosuppression is associated with a dysbiotic state characterized by overgrowth of *Candida* and certain resident bacterial species. Bacteria may interact with *Candida* species to augment the mucosal inflammatory response, resulting in epithelial damage. Alternatively, bacteria can directly influence filamentation and virulence gene expression to increase mucosal invasion. PRR: pattern recognition receptors; TLR: toll-like receptors; NOD: nucleotide-oligomerization domain.

Polymicrobial infections with *C. albicans* and bacteria are not only more prevalent, but also more lethal in immunosuppressed patients [82,83]. We propose a pathogenesis model whereby immunosuppression coupled with *C. albicans* colonization results in a bacterial dysbiosis with dominant species that have the ability to promote fungal virulence, thus acting as synergistic pathobionts (Figure 1). However, studies exploring shifts in the mucosal microbiome during pharmacologic immunosuppression and fungal infection in humans are not available and are sorely needed. Immunosuppression can have a detrimental role in shaping the local microbial environment where *C. albicans* infections occur. For example, a study of the colon microbiome in transplant patients receiving immunosuppressants showed major shifts with a predominant increase in the proportion of enterococci and a decrease in other *Firmicutes*, starting in the immediate post-transplant period when these patients are also more susceptible to gastrointestinal candidiasis [84]. Interestingly, in cases of graft-versus-host disease, this shift was even more pronounced, raising the possibility of enterococci acting as pathobionts in this clinical setting [84].

In conclusion, the pathogenesis of mucosal candidiasis is integrally related to the physiology of the resident microbial communities within which *C. albicans* adapts to either colonize or cause disease. Resident bacteria can directly modulate the virulence of *C. albicans* by altering virulence gene expression, or indirectly by modulating the host response. Therefore, “niche-specific” fungal responses to the local microbiota and reciprocal effects on mucosal bacteria are an important component of fungal pathogenesis in non-sterile sites. In future studies, mucosal infection models should take advantage of the knowledge gained from the metagenomics field to identify resident commensals with the potential to become pathobionts and define mechanisms of pathogenic synergy with *Candida*. These studies will pave the way for better preventative and therapeutic strategies in mucosal candidiasis.
